# Stretchable Sensor Materials Applicable to Radiofrequency Coil Design in Magnetic Resonance Imaging: A Review

**DOI:** 10.3390/s24113390

**Published:** 2024-05-24

**Authors:** Rigoberto Vazquez, Elizaveta Motovilova, Simone Angela Winkler

**Affiliations:** 1 Department of Biomedical Engineering, Cornell University, Ithaca, NY 10065, USA; 2 Department of Radiology, Weill Cornell Medicine, New York, NY 10065, USA

**Keywords:** soft substrates, liquid metals, fabric textiles, 3D printing, stretchable sensors

## Abstract

Wearable sensors are rapidly gaining influence in the diagnostics, monitoring, and treatment of disease, thereby improving patient outcomes. In this review, we aim to explore how these advances can be applied to magnetic resonance imaging (MRI). We begin by (i) introducing limitations in current flexible/stretchable RF coils and then move to the broader field of flexible sensor technology to identify translatable technologies. To this goal, we discuss (ii) emerging materials currently used for sensor substrates, (iii) stretchable conductive materials, (iv) pairing and matching of conductors with substrates, and (v) implementation of lumped elements such as capacitors. Applicable (vi) fabrication methods are presented, and the review concludes with a brief commentary on (vii) the implementation of the discussed sensor technologies in MRI coil applications. The main takeaway of our research is that a large body of work has led to exciting new sensor innovations allowing for stretchable wearables, but further exploration of materials and manufacturing techniques remains necessary, especially when applied to MRI diagnostics.

## 1. Introduction

**Figure 1:**
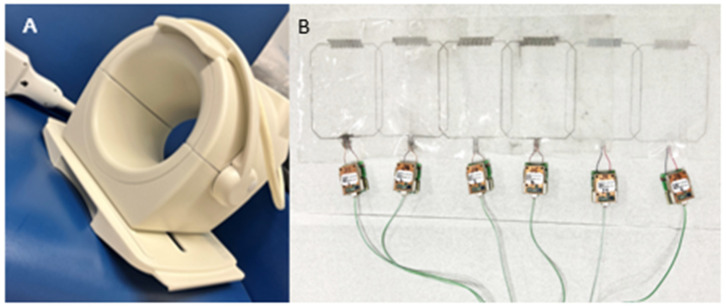
(**A**) The 8-channel flexible knee array coil; (**B**) 6-channel stretchable polymer knee array coil [13].

Each year, 40 million individuals undergo magnetic resonance imaging (MRI) scans in the United States [1]. MRI technology aids in differentiating soft tissues and is used to help diagnose diseases, monitor treatment, and guide therapeutics. An essential component of MRI hardware is the radiofrequency (RF) coil, which induces and detects rotational magnetization in tissue, resulting in an MR signal via electromagnetic induction that subsequently allows for the creation and construction of high-quality images. The intricacies in RF coil designs impact spatial and temporal resolution as well as uniformity and overall quality of MR images. Typical RF coils are built from rigid materials and made to image a dedicated anatomical region, such as the head, knee, and wrist (see Figure 1A for a knee coil example). Although such RF coils are designed to fit the general population, the wide variability in patient anatomy, in particular their body dimensions, can influence the efficiency of the coil, which directly impacts image quality. This is because the signal-to-noise ratio (SNR) of the imaging signal decreases with the distance between the coil and the tissue [2], resulting in suboptimal SNR when a coil is far removed from the surface of the anatomy and therefore poorly loaded. Lower SNR leads to a reduction in image resolution and diagnostic quality or results in lengthy scans, thereby impacting cost and patient throughput. In addition to these performance disadvantages, rigid RF coils can also cause patient discomfort, especially during prolonged exams [3], which can ultimately result in reduced patient compliance. In response to these challenges, the state-of-the-art MRI RF coil design has focused on maximizing SNR, demonstrating consistent performance across a wide range of patient populations, and providing suitable patient comfort.

To address these limitations of traditional MR RF coils, increased research emphasis has been placed on the design of flexible RF coils (see Figure 1B for an example of a stretchable knee coil) that can accommodate anatomical variations and complex curvature (e.g., groin, chest, breast). These new concepts sometimes even provide dynamic imaging capabilities and allow the movement of the anatomy without limiting image quality [3].

To design such stretchable MRI coils, unconventional materials and solutions that conform to the anatomy of interest are needed. In particular, copper wires are being replaced by alternative conductive materials, such as liquid metals [4] and conductive threads [5]; typically used printed circuit boards (PCBs) are being replaced by stretchable polymers [6] and textiles [7]. Nonetheless, suitable material selection providing optimized elastic capabilities, sufficient electrical conductivity, and adequate biocompatibility has proven to be quite challenging. While liquid metal ink-based composites possess many favorable, unique, and superior structural characteristics, fabrication processes must be established and optimized [8,9]. For instance, in 3D printing-based manufacturing, process variables must be optimized and modified [10,11] for different conductive inks to ensure functionality and performance.

Beyond the selection of substrate and conductor materials, it is also necessary to develop new technology for lumped elements such as capacitors. From a sensor perspective, RF coils are traditionally composed of a conductive wire loop, typically made from copper, which serves as an inductor, and capacitive components, which are usually lumped elements [12]. The capacitance and inductance determine the resonance frequency of the “sensor” coil, at which it can pick up the MR signal from the anatomy. This signal represents the precessional behavior of spins in the body and resonates at the Larmor frequency, varying proportionally with the static magnetic field strength $B_{0}$ (128 MHz at 3.0 Tesla (3T), 298 MHz at 7.0 Tesla (7T)). Implementing the capacitances in coils that are flexible or even stretchable requires innovative solutions that break away from traditional lumped element components.

Finally, the combination of novel materials and circuit components also requires the development of adequate manufacturing methods.

Although the research on stretchable sensor technology has progressed and a range of prototypes have been reported, it is safe to state that material selection and lumped element development for specific complex anatomical applications is still in its infancy, especially when applied to the area of MRI RF coil development.

The objective of this paper is to review the literature on existing flexible sensor solutions with a particular focus on MRI coil applications. The review encompasses the following:

(1) A systematic overview of existing flexible/stretchable RF coil technologies;
(2) A review of stretchable substrate materials usable in sensor designs;
(3) A summary of stretchable conductive materials;
(4) An overview of the pairing of these substrates and conductors to form a sensor element;
(5) A section on the implementation of lumped element capacitors;
(6) A review of applicable fabrication methods;
(7) A brief commentary on the use of these sensor technologies in future MRI coil applications.

We also discuss the use of fabric materials in each section as textiles are becoming more and more popular in the design of wearables and RF coils.

## 2. Recent Development in Flexible MRI RF Coil Technology

Recent advances in the design and development of RF coils have been driven by the desire to optimize SNR and improve patient experience. This research has resulted in a number of flexible and stretchable solutions [3].

### 2.1. Flexible Coil Designs and Related Requirements

Several research groups reported having successfully designed and developed flexible RF coils based on coaxial cables [14], screen-printed arrays [15,16,17], liquid metals [8,13,18,19,20], and conductive polymers [21]. Major MRI vendors have also made significant efforts to produce commercially available lightweight coils that apply less pressure onto the patient compared to previous coil generations (AIR^TM^ Coils from GE Healthcare [22], Contour Coils from Siemens [23], and Shape Coils from Canon [24]). Research prototypes range from rigid-adjustable [25,26,27] to flexible [28,29] and even stretchable [8,21,30,31] solutions. The degree of flexibility of a coil prototype depends on the design approach and the choice of coil materials. Several semi-rigid coil arrays have been proposed that can be wrapped around a 2D curved surface [32,33,34,35,36,37,38,39] or mechanically adjusted to better fit the targeted area [25,29,32,40,41], thereby enabling the accommodation of different patient sizes. Coaxial transmission line resonators, also called “coaxial coils”, “cable coils”, and “high-impedance coils”, have recently gained new momentum. Although the idea of using coaxial cables as flexible MRI coils dates back to 1987 [42], several research groups characterized and demonstrated new coil designs based on this technology [43,44,45,46,47,48]. Moreover, the light weight, flexibility, and ease of manufacturing of such coils allow for the simplified construction of modular coil arrays for multi-purpose applications [49].

### 2.2. Lumped Element Integration

Implementation of lumped element capacitors in flexible designs can be challenging. Coaxial cable coils, when used as distributed resonators, do not require lumped elements along the coil conductor, relying instead on the precise separation between the inner conductor and the outer shield for the required capacitance. Cable coils are thin and flexible and offer improved inter-element isolation, enabling dynamic imaging applications [43,44,46,47,50]. In [51], a thin and flexible coil was printed on both sides of a Kapton substrate to create the required capacitance in distributed form for coil resonance. The screen-printed coil fabrication method developed by Corea et al. [15,28] avoids bulky copper wires, porcelain capacitors, and thick substrates, allowing for more flexibility and closer coil placement, which is especially suitable for pediatric imaging [16].

### 2.3. Stretchable Designs and Frequency Retuning

Although these designs offer high flexibility, truly stretchable coil designs require the use of unconventional conductive materials such as copper braids [52], conductive thread [30,53,54,55], liquid metal [8,18,21,56,57,58], and conductive elastomer [31]. Even though such materials are lossier than traditionally used copper wires, it was shown that they can offer similar SNR and provide additional advantages such as light weight and radiological transparency [59]. Moreover, stretchable designs experience frequency detuning because the resonator inductance increases with stretched conductors. This represents one of the biggest roadblocks incurred in the reliable development of stretchable coils. Several solutions have been proposed to mitigate this frequency shift, including field programmable gate array (FPGA)-based tuning/matching circuits [60], π-matching networks [40], and an inherently self-tuning coil geometry [8].

### 2.4. Biocompatibility and Patient Safety

When using novel and alternative materials for MRI RF coil development, it is important to not only ensure adequate coil performance but also patient safety. Thus, the nontoxicity and biocompatibility of materials for coil conductors and substrates are of high concern. For example, an early research coil prototype used mercury liquid metal [57], a toxic chemical that poses high safety risks if accidentally spilled. Currently, liquid metal-based flexible coil prototypes [8,21,61] use eutectic gallium indium (eGaIn), which remains liquid at room temperature, has low viscosity, and is considered to be non-toxic [62].

### 2.5. Materials with Optimized Electromagnetic and Mechanic Performance

Using conductive polymers [31] can prevent potential liquid metal spillage but comes at the cost of reduced electrical conductivity. Conductive threads offer rapid and consistent manufacturing using a professional embroidery machine and provide greater tensile strength than 30 AWG copper wire [30,63]. Substrate/encapsulation materials vary from silicone tubes [21] and polymer microfluidic channels [8] to stretchable fabrics [64].

Although the demonstrated wide range of flexible and stretchable conductive materials and substrates used to construct MRI receive coils shows intriguing results that each comes with specific advantages and disadvantages, there is an even wider range of recently developed materials and fabrication techniques emerging in the broader field of stretchable sensors. The following sections will review these novel flexible sensor designs and materials and examine their potential translation into MRI research.

## 3. Stretchable Substrate Materials for Sensors

**Figure 2:**
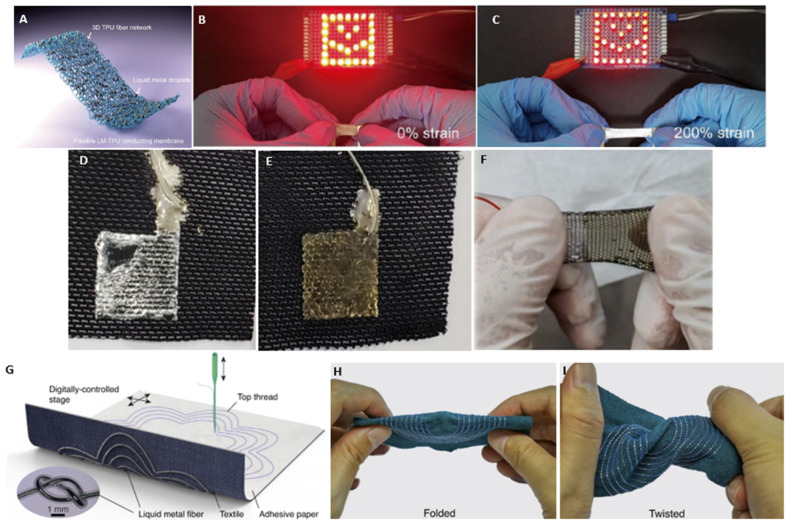
(**A**) Flexible liquid metal-TPU composite film (**B**) connected to a smiley face pattern to show light intensity variation under 0% strain and (**C**) 200% strain [66]. (**D**) Liquid metal particles (LMPs) sprayed on treated stretchable fabric substrate. (**E**) Gold nanoparticles (Au NPs) deposited on LM fabric. (**F**) AuLMPs textile sensor under stretch [64]. (**G**) Embroidered textile sensor subjected to (**H**) folding and (**I**) twisting [69].

Many design limitations inherent to stretchable sensors stem from a fundamental understanding of material handling, surface adhesion, and selection mismatch. To further the development of flexible sensors in general and stretchable MRI coils in particular, it is crucial to revisit current advances and challenges in established substrate materials such as elastomers, glass, hydrogels, and thermoplastics. There are many potential materials to be fully explored. Some of the most common stretchable sensor composites consist of thermoplastic polyurethane (TPU) or rubbers such as polydimethylsiloxane (PDMS). These materials have been studied extensively for their benefits in terms of elasticity, dielectric permittivity, optical transparency, biocompatibility, and biostability [65,66]. An example of a stretchable sensor composite can be seen in Figure 2A–C.

However, some of the disadvantages associated with these composites are their need for additives (i.e., carbon grease or single-walled carbon nanotubes) and the need for expensive post-processing (i.e., electrospinning) [67]. In particular, carbon grease is highly viscous and has been shown to cause high hysteresis in stretchable sensors due to the lack of self-healing capabilities while also causing material resistance under elastic deformation [68].

Stretchable electronics derived from films, plastics, or ceramics can retain their properties under deformation are optimal. For instance, thermoplastic copolymers—e.g., Dragon Skin silicone (Smooth-On, Inc., Macungair, PA, USA) and Ecoflex silicone (Smooth-On, Inc., Macungair, PA, USA), have emerged as potential solutions to address many of the mechanical challenges associated with flexible sensors. These thermoplastic materials have shown good elasticity and tensile recovery under extensive cyclic loading. Ultimately, substrate materials must be substantially optimized in a multi-step process for full electromechanical functionality in wearable sensor designs.

### 3.1. Structural Characteristics of Fabrics Suitable as Substrates

Modern electronic textiles are moving towards two-dimensional planar materials, such as woven (i.e., fabric interlaced with two or more threads) and non-woven (i.e., entangled filaments) fabric, and have been used in a range of applications such as electromagnetics [70], antennas [71], tubular fiber-based wearable electronics [72], energy harvesting cloth [73,74], and monitoring devices for biomedical vital data [75].

The main differences that categorize conventional textiles are that woven fabrics have a higher elastic modulus, while non-woven fabrics exhibit low elasticity and a higher permittivity [76]. Other highly sought out textiles are categorized by their manufacturing process, such as knitted, spinning, and mono- and multifilament element braiding. These have shown great potential for textile substrate sensor applications [77]. In particular, the knitted fabric offers formidable stretchability (as seen in Figure 2D–F) but has shown a mechanical recovery loss of ~166%, and thus comes with limitations to geometry and stretching [64]. A detailed overview of textile manufacturing processes and materials can be found in [78].

Textile substrate microstructure should also be considered prior to substrate selection. Some fabrics exhibit advantageous microstructural features leading to beneficial mechanical properties. However, most fabrics must undergo some form of modification along the lines of specific coating or mass-doping to aid in preventing tearing and conductive filler ink percolation through the fabric [69,79,80]. The resulting increase in conductive filler content in stretchable substrates provides a thick device that can greatly affect the elastic properties of the conductive sensor [81]. Therefore, a compromise must be made between mechanical performance and the desired electronic functionality of the sensor when selecting a substrate.

In general, hydrophobic textiles are preferred in sensor applications, as water in fabric substrates reduces the resonance frequency [82]. Most recently, woven cotton textiles have been considered in many medical devices due to their hypoallergenicity and permeability. Researchers have demonstrated dielectric substrates made from cotton and polyester with conventional copper wires mounted on a stretchable patch antenna, resulting in a reasonable resonance frequency stability of ~6% [83]. Cotton-based textiles have been shown to have high thickness, low density, and high resistance. However, these textiles can exhibit a rough structure as well as a hairy and porous surface [84]. Polyester substrates have a lower thermal conductivity with higher elongation [85]. Polyester substrates have been shown to exhibit an optimal printing direction, allowing for maximized performance when certain geometrical constraints are observed.

Recently, efforts have been made to improve upon or to completely replace conventional dielectrics with textile-based substrates to optimize their electromechanical characteristics, breathability, and high load deformation durability in wearable sensor applications [72]. Fabric-based textiles have been known to serve as dielectric substrates in a wide variety of sensor applications (i.e., “e-textiles”) [82]. Table 1 shows a comparative analysis of conventional dielectric textile materials to aid in the substrate selection process [86], consisting of dielectric constants, tangent loss, and other parameters crucial in electric and electromagnetic sensor applications.

Optimized substrate material selection is necessary as the capabilities for sensors are theoretically infinite. One of the key parameters determining the electromagnetic performance of a substrate is its dielectric permittivity and loss tangent. The textiles in Table 1 represent substrate options with low relative permittivity and low loss tangent.

### 3.2. Integration of Polymers with Fabric Substrates

In addition to the standalone use of fabric, researchers have begun to exploit novel material combinations by integrating polymers with textile substrates. This allows for formidable surface consistency, enhanced fabric durability, prevention of wear and tear, and better control of the dielectric permittivity of the substrate. For instance, textile yarn fibers have been coated with PEDOT:PSS in order to allow for 1D linear sewing and 3D spatial embroidery techniques [78].

It is important to continue this pairing of different polymeric materials with a variety of textile substrates to further explore the potential behavioral characteristics of flexible sensors and to ensure the mitigation of unwanted adverse skin sensitivity reactions, such as contact dermatitis. For instance, increasing the hardness of the fiber composite core can aid in preventing large tearing or puncturing but comes with reduced flexibility of the textile [69], which can be seen in Figure 2G–I, potentially leading to destroyed electrical connections of the liquid metal.

Prior to selecting a suitable material composite, it is paramount to optimize its electromagnetic characterization. While there is limited information on the dielectric properties of normal fabrics, the electromagnetic behavior is easier to characterize in polymer-fabric composites.

### 3.3. Biocompatibility of Substrates

Ideal substrate candidates need to be tested for their toxicity and biocompatibility. Not only are the electrorheological properties of the material crucial for the wearable sensor, but the potential biological effects of sensor materials when interacting with epidermis tissues need to be studied in detail.

Novel materials can exhibit promising performance but are often harmful to the human body. Among the examples are recently proposed substrates with additive semiconductive materials such as single-wall carbon nanotubes (SWNTs) and ceramic-based nanoparticles such as BaTiO_3_ [88]. Carbon-based nanoparticles [89] or substrates doped with barium compounds [90] have been shown to exert toxic effects, and more research must be conducted when implementing and pairing these metal-ceramic-based material formulations.

Recently, non-hazardous materials have gained attention. For example, poly(vinyl alcohol) (PVA), nanocellulose fibers, and starch or amylum (AM), have been found to contain desirable features for use in substrates [91].

## 4. Stretchable Conductors Materials for Sensors

**Figure 3:**
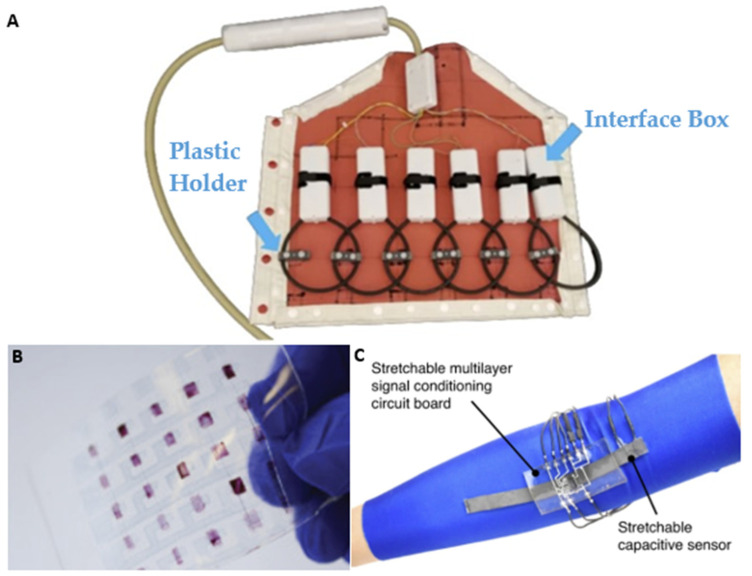
(**A**) Custom rigid cable coil conductors for a knee coil array sensor [36]. (**B**) An elastomeric conductor within a stretchable patterned electrode for a sensory skin system [92]. (**C**) Interfacial component connection of an elastomeric conductor and flexible (or rigid) conductor within a stretchable circuit [93].

A stretchable sensor requires a conductor that can elongate with the increase in sensor size. Thus far, in the literature, such “stretchable” conductive materials include both emerging material formulations (i.e., liquid metal, natural resins, and metamaterials) and conventional materials (i.e., copper, silver (Ag) flakes, graphene, and carbon-based materials). Alternative, “stretchable”, conductor materials that can be used in flexible electronics must have sufficient electrical conductivity to maximize SNR and sensor sensitivity [36] (see Figure 3A). In the following section, we specifically examine the electrical conductivity of different stretchable conductors.

### 4.1. Electrical Conductivity of Stretchable Conductors

Conductors with higher resistivity increase the losses of the coil per se. In MRI, SNR is increased when the sample loss dominates over coil loss. Therefore, it is usually a design goal in MRI coil development to minimize coil losses as much as possible as the unloaded-to-loaded Q-factor ratio will be maximized. In our prior work with eGaIn embedded in elastomer, we measured a resistivity of 13.8 × 10^−8^ Ω·m, which resulted in an unloaded-to-loaded Q-factor of 2. This has proven sufficient for the proper performance of our coil [8]. As a result, the SNR gain from a highly conformal fit and thereby minimized distance to the anatomy, along with maintaining the coil resonance frequency in its stretched state, has had a much larger impact than the minimal concern from slightly increased conductor resistivity. The influence of conductor resistivity is an important area of research and needs to be investigated in detail in isolation in order to provide an adequate estimation of the overall change in SNR for wearable coils. Various materials have been proposed to aid in improving the electrical performance of wearable sensor systems. Table 2 shows a summary.

### 4.2. Conventional Conductors

Many of the commonly used conventional conductive materials, such as copper, Ag, and conductive polymer composites, as found in Table 2, can be integrated into flexible substrates. Traditional wiring materials (e.g., copper, aluminum, and gold) are highly conductive but rigid and strain-limited, which impacts their electrical performance under deformation.

### 4.3. Liquid Metals

Liquid metal composites have been proposed to improve the ease of integration with different materials and have been used in many applications, such as antennas [106,107], metamaterials [108], interconnects/interfaces [109,110], elastic wires [21], and electrodes of various shapes [111,112,113], as well as different inductive and capacitive sensors [114,115,116]. Typical liquid metals used are mercury, francium, cesium, rubidium, gallium, and its alloys such as eutectic gallium indium (eGaIn) and gallium indium tin (Galinstan). Of these materials, eGaIn appears to be among the most promising.

However, gallium (Ga) and its alloys undergo surface oxidization when exposed to an ambient setting. As a result, liquid metal interfacial bonding forces prevent direct adhesion with the substrate and the liquid self, reducing the electrical conductivity during elastic testing. In past studies, acid treatments or applications of an electric field have been used to overcome the bulk state of the oxide layer [113]. However, when removing the oxide layer in this manner, the liquid metal converts to a bulged shape, which can inhibit electrical conductivity within the composite [117].

### 4.4. Material Composites and Additives

A big focus has been placed on introducing additives to conductive materials in order to increase electrical performance. A few groups have pursued the rheological characteristics of liquid metals by adding solid-phase materials to aid in improving electrical conductivity. Huang et al. made substantial progress and demonstrated the embedding of liquid metal in polymeric-based microfluidic antennas with a complex geometric serpentine shape [118]. The device achieved a stable resonance frequency and stretching capability of up to 50%. Other groups, e.g., Wang et al., reported low sensitivity under stretch in liquid metal-based microfluidic gauge sensors and suggested embedding SiO_2_ microspheres [119]. On the other hand, reports have shown that such additional materials (i.e., metallic and non-metallic particles, native oxide flakes, or even a mixture of two or more of these materials) overall do not provide enough fluidity for proper dispensing during fabrication [120,121] as they develop a crumpled form that hinders continuous electrical conductivity [122,123]. Research has also centered around the use of conductive polymer materials, such as poly(3,4-ethylenedioxythiophene)–poly-styrene sulfonate (PEDOT:PSS) and poly(3-hexylthiophene) nanofibers (P3HT-NFs)/PDMS, to improve conductivity. A rubbery conductor using a flexible PEDOT:PSS-based film was fabricated on a stretchable PDMS substrate (see Figure 3B) [92]. The same group made an additional separate semiconductor that delivered optimal rubber-like properties by using a P3HT-NFs/PDMS composite. Both rubbery strain sensors provided a high gauge factor, formidable conductivity, and reliable temperature performance [92].

Some groups have opted to use alternative materials to improve conductivity, including specific form designs of thin metal films [124,125] and carbon-based materials [126]. Carbon-based and graphene composite materials are interesting candidates for sensor wiring due to their high conductivity of 1.5 × 10^4^ S/m and applicable flexible stretchable properties [127,128]. Xu et al. synthesized a graphene-based fiber composite material doped with silver ions to prepare a highly conductive flexible stretchable fiber [129]. The Ag-doped graphene fibers possess an astounding electrical conductivity of up to 9.3 × 10^4^ S/m. However, just like many other similar conductive materials, graphene has been reported to exhibit issues related to stability, dispersion in liquids, and temperature annealing, ultimately hindering integration in wearable sensors [130].

Others have sought out biphasic-solid composites, for instance, high-conductivity materials (i.e., Ag nanowires (NWs)) integrated with other filler electrode material (i.e., PEDOT: PSS), to better increase both mechanical and electrical properties in flexible sensor substrates [131]. An illustration of a similar biphasic composite conductor can be seen in Figure 3C [93].

Many of the proposed additive materials are promising and efficient for use in wearable devices. However, some have yet to be explored. In particular, silicene [132], used in a sensor with a low gauge factor, has yet to be fully examined but is expected to supersede graphene in flexible sensor design as it has good adhesive properties, high conductivity [133], and excellent elasticity [134]. Ultimately, it is important to note that the incorporation of certain additive materials can reduce conductivity in the composite ink [135] as illustrated in Table 2. This is likely due to the ratio of Ag NWs vs. liquid metal in the composite. Conductive filler composites aggregate within the surface substrate and tend to disrupt the fluidity of the conductive network when undergoing stretching [136].

### 4.5. Conductive Fabrics

As an innovative alternative, researchers have also begun to explore the integration of conductive materials within stretchable textiles due to their low dielectric permittivities as well as their anisotropic and compressible material characteristics [82]. A conductive nylon fabric coated with polymeric substrates was used to construct a tunable frequency wearable antenna but ultimately resulted in load cycle deformation issues [137]. Therefore, further exploration of new materials while taking secondary effects into account must continue in order to push performance boundaries.

### 4.6. Biocompatibility of Conductors

While many of the available and aforementioned conductive inks and other materials are encapsulated or embedded within specific substrates, emphasis must be placed on studying their inherent toxicity.

Of the conductive materials mentioned thus far, biological effects on tissues have been discussed in a variety of studies [138,139,140]. The most common conductive material used is copper, known to have anti-inflammatory properties and immunogenicity with dermal exposure. However, copper has been shown to oxidize and degrade when exposed to the acid components found in sweat [138]. While many methods have been proposed to overcome copper oxidization, it is still a rigid material and not malleable enough in cold temperatures to lend itself to flexible sensor development.

Instead, liquid metal has been commonly used in flexible devices as a non-toxic substitute for toxic liquid mercury. However, some substrates such as fabrics are weakly adhesive with liquid metal, making conductive patterning difficult [141].

Recently, styrene block copolymers have emerged as a potential additive material to increase adhesion (see Section 5, where we discuss such additives); however, styrene-based composites have been shown to result in toxicity [142,143]. Iron has been sought out as a better, non-toxic, solid-phase additive material due to its favorable conductivity, embedding flexibility, and biocompatibility.

## 5. Considerations When Combining Substrates and Conductor Materials

The development of flexible sensors requires fundamental knowledge of the dielectric parameters for the substrate (i.e., permittivity and thickness) and parameters for the conductive ink (i.e., printing direction, filler electrodes, and substrate capability properties) [144]. Moreover, the adhesion between substrate and conductor is of crucial importance.

### 5.1. Ensuring Adequate Adhesion

One of the most important considerations in combining substrate and conductor materials is their adhesive compatibility. The need for an additive material to enhance the adhesion of liquid metal or other conductors to a highly stretchable and flexible substrate is paramount.

### 5.2. Additive Material Innovations That Increase Adhesion

Some of the concepts used to improve conductivity in conductors have served to increase adhesion to their substrates simultaneously.

In [145], an Ag-In-Ga-poly(styrene-isoprene-styrene (SIS)) ink composite with added styrene–isoprene block copolymer enabled better mechanical integrity and adhesion on many substrates such as rubber and textile materials. Styrene block copolymers (i.e., SIS and poly(styrene-butadiene-styrene) (SBS)) have been integrated into conductive inks and exhibit great elasticity, conductivity, and adhesive properties, making them interesting additive candidates to be paired with liquid metal [146]. However, many of these styrene resins have a potentially hazardous effect on the skin (see Section 4.6), requiring further analysis.

Luo and Zhou reported having successfully prepared a flexible conductive ternary composite consisting of silicone, liquid metal, and iron particles, resulting in a composite system with superior elastic performance, excellent repeatability, microstructure stability, formidable conductivity, and high breaking elongation [147].

**Figure 4:**
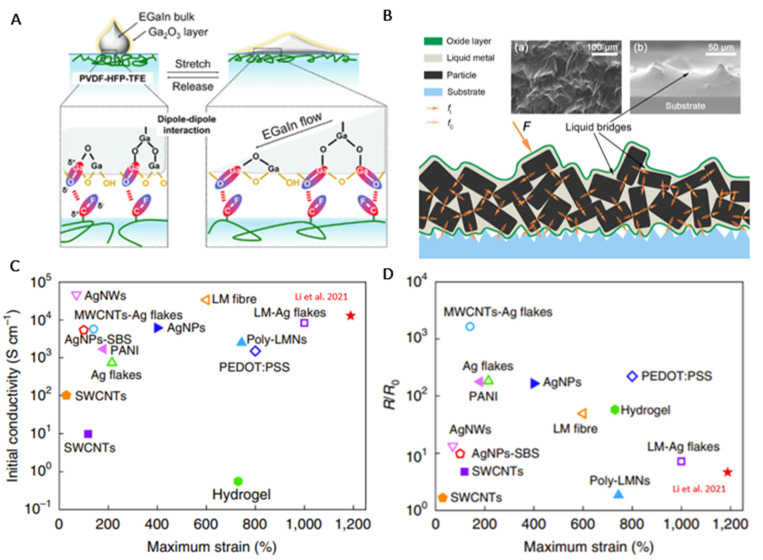
(**A**) Schematic illustration of the behavior of stretch−release of eGaIn on a substrate [154]; (**B**) depiction of biphasic composition framework [155] ((**a**) rugged surface of TransM^2^ixes induced by solid particles (top view) and (**b**) liquid bridges formed between solid particles (cross-section view)); (**C**) relative resistance change at initial 0% strain for stretchable conductors composites [93]; (**D**) relative resistance change at maximum strain for stretchable conductors composites [93].

Several research groups have also implemented acidic resins in the ink to improve liquid metal interfacial adhesion (Figure 4A, eGaln bulge formation) [113]. While natural resins such as tannic acid [148] or fructose [149] can improve liquid metal interfacial adhesion capabilities, many of these acids undergo degradation of liquid metal and thus exhibit low structural stability [150]. A few groups have also explored cellulose. In particular, a Ga^3+^ ion layer on the liquid metal that ionically crosslinks with the hydroxide ions of cellulose hydrogels was demonstrated and showed significantly improved electrochemical properties [151], suggesting that natural resins have potential in a wide range of wearable biomedical applications.

In a new area of interest, Ochirkhuyag et al. provided an extensive review of the combinations of biphasic liquid metal mixtures (i.e., alloyed micro- or nanoscale rigid metals mixed with liquid metal) in elastomeric substrates for a variety of sensor applications [152]. The review further provided insight into the advantages of biphasic liquid metal mixtures in terms of their good adhesion to various substrates. An illustration of a biphasic composition framework is shown in Figure 4B. Another example of a biphasic composite used to manufacture a printable circuit board was developed in [93]. In this example, the stretchable sensor was made by incorporating crystalline solids in biphasic eGaIn filler material on a silicone/very high bond (VHB) tape substrate. The device achieved high stretchability (~1000%) and excellent conductivity (~2 × 10^6^ S/m), did not exhibit resistance under deformation, and showed great interface compatibility with rigid composites. Liu et al. went on to compare the initial and maximum strain resistance of their sensor to the resistance change in other proposed rigid materials as illustrated in Figure 4C,D. A mixture of an ionic-covalent alginate/polyethylene glycol/polyacrylamide crosslinked hydrogel was assembled in [153], enabling digital printing and formidable adhesion. Ultimately, their sensor system proves to be superior to many of the conventional materials. Herein, Table 3 presents a range of substrate and conductor composites specifically designed for flexible and stretchable sensor applications.

**Table 3:** Summary of processes utilizing mixtures for material compositions in common sensor applications.

	Applications	Materials	Fabrication Methods	Ref.
**Polymer** **Sensors**	Stretchable circuit board	LMs/Crystalline solids/Silicone	Spray printing	[93]
	Stretchable circuit	Ag-InGa-SIS	Printing	[145]
	Flexible sensing patch	Silicone-LMs-Fe	Molds	[147]
	Stretchable electrode	LMs/Graphene/Ecoflex	Direct Writing	[150]
	ECG	LM-particles/cellulose	Stirring	[151]
	Soft-matter system	LMs/Ag/SIS (ink) + alginate/PEG/PAAm (substrate)	Printing	[153]
**Fabric** **Sensors**	Wireless interconnection	Garment/LM/PFA-polymers	Embroidery	[69]
	Multi-layer electronics	Polyethylene terephthalate (PET)-textile/Ag/UA/PVP/PMF	Inkjet-Printing	[156]
	Soft-matter system	TPU/LM/NFs nanoyarn	Electrospinning	[157]
	EMG System	CW-X-Textile/Ag flakes/fluorine rubber/Fluorine-surfactant	Stencil printing	[158]
	Electronic textile	LM/tube/CNT polyester yarn/PET fiber	Friction spinning	[159]

### 5.3. Combining Stretchable Conductors with Fabric-Based Substrates

A few groups have begun exploiting conventional conductor materials for use with fabric substrates. Many of these materials are highly conductive, affordable, and easy to integrate into textile substrates without the need for complex fabrication techniques (i.e., embroidery, and sewing) [94]. However, these conductors are usually not stretchable, only flexible.

Prior to combining fabric-based substrates with stretchable conductive inks such as liquid metal or its composites, the correct materials have to be considered. The unique features of textiles such as their density, elongation, material composition, weave, thread diameter, surface characteristics, and capacity capabilities need to be considered. Further, conductive features should have solid ink content and low viscosity, as they can penetrate or be absorbed within the threads of textiles [144].

#### 5.3.1. Depositing Larger Conductive Elements on Fabric Substrates

A successful selection of textile materials should not only depend on the intended sensor application but also on the most feasible way to adequately implement proper electrode patterning. Implementing larger conductive areas such as electrodes in textiles can be a complex challenge. The Young’s modulus, electrode mobility, conductivity, distance sensing sensitivity, and permittivity [94] of the resulting sensor can suffer when the wrong materials are paired. Therefore, prior to assemblage, it is crucial to understand (i) the inherent anisotropic microstructure of the textile, (ii) the electromechanical performance of the porous textile substrate, and (iii) the changes in (i) and (ii) after encapsulation of conductive electrode fillers within woven fiber voids.

#### 5.3.2. Liquid Metal Additives Providing Adequate Electrode Patterning

Adequate pairing of liquid metal with suitable materials aids in stabilizing the conductive filler structures once embedded in the textile substrates. Various methods have been explored to obtain such textile-stable conductive inks. For instance, Zhu and Cheng reviewed strategies used to integrate conductive polymer composite (e.g., polyurethane, Carbon nanotubes(CNT), graphene) fillers and solid-phase metals (e.g., nickel, Ag, and copper) [83]. Many of these composites can be further modified to form conductive elements within a wide variety of sensor substrates [160,161,162,163,164]. In this realm, some researchers have paired conductive polymer composites (i.e., PEDOT:PSS) with other electrode filler materials (i.e., liquid metal) to improve conductivity within the assembled stretchable sensors [165]. However, many of these devices show minimal strain endurance under deformation. Adequate pairing and structural stabilization of electrode materials need to be examined further.

**Figure 5:**
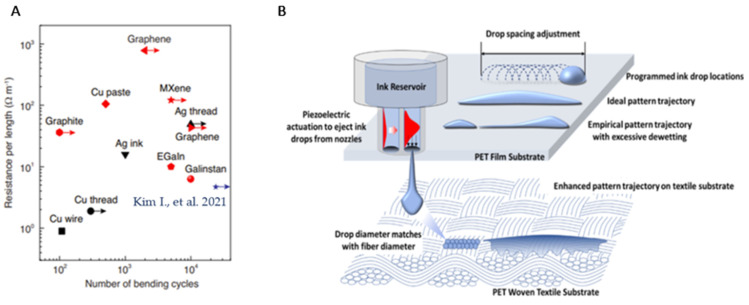
(**A**) Comparison of conductive materials used for textile (black color) or flexible (red color) electronics under deformation [69]; (**B**) printing process and ink spreading behavior on textile substrates [156].

As discussed earlier, conductive inks have been made or derived from carbon nanotubes, Ag, conductive polymers, or even graphene, as illustrated in Figure 5A. In many of these existing conductive materials, an inherent tradeoff had to be made in their electromechanical properties in order to allow for their use in textile substrates [69]. Among these drawbacks is the nonuniform dispersion of filler material within the substrate, leading to aggregates and compromised overall mechanical performance [166]. On the other hand, some researchers show that coated conductive textile fabrics underperform when using fabric-based textiles with conductive fibers due to unevenness of the substrate (i.e., surface resistivity) [82]. The addition of these materials to the electrode filler network can also lead to low electromechanical stability [167].

The combination of textile and conductive materials has also been investigated in [69]. Matsuhisa et al. 2015 reported a flexible wearable conductive sensor printed on a textile substrate with functional ink composed of Ag flakes, fluorine rubber, and fluorine-surfactant composites. The delamination of electrode filler networks embedded in the rubber matrix was overcome via the addition of adhesive agents to the ink formulation [158,168].

#### 5.3.3. Optimizing Electrical Conductivity via Additives in Fabric-Based Substrates

As described earlier, most conductive materials in existing composites contain add −itives such as Ag NWs, carbon nanotubes, and graphene to improve electrical conductivity [169]. In order to provide similar conductivity improvements in conductive electrode fillers within textile substrates, researchers have used fabric combined with metal-coated yarns, nanowires, conductive polymers, carbon materials, ceramics, and semiconductors, as well as organic and inorganic materials [167].

Thus far, the pairing of textiles with conductive fillers has sometimes led to rigid materials and conductive loss (see schematic for ink behavior on textile fabric in Figure 5B). Exposing skin to uncovered liquid metal or electrode fillers, even if non-toxic, can also lessen conductivity [157]. While efforts to embed or encapsulate such conductors within the textile seem to be the solution, this often leads to a bulky device. Many research groups have focused on optimizing the process parameters of the textile surface chemistry through printing, annealing, and other methods [156]. Recently, an embroidered wireless system consisting of perfluoroalkoxy alkane tubing infiltrated with liquid metal fiber has been designed [69]. Similarly, a sensor was built using thermoplastic TPU/Ag flake fiber coated by a layer of waterborne TPU, followed by a liquid metal coat [170]. This conductor was embedded within a PDMS composite fiber through wet spinning and showed a stable balance of stretchability and electrical conductivity for the design fibers, in addition to a continuous conductive path within the fiber core necessary for rigid materials. While progress has been made with regard to using liquid metal conductive alloy core threads (i.e., fibers) on woven fabrics, mismatches in materials and complex manufacturing techniques have hindered sensor sensitivity and durability, ultimately making them incompatible with threads made from yarns or polyester textiles [159,171].

Selection of the most compatible substrate/conductor material combination, as well as usage of the best-suited textile fabrication process, is essential in the optimization of wearable sensors. To ensure conductivity throughout voids within the textile substrate, the proper selection of electrode fillers, surface chemistry modification, and use of material additives with the necessary electrical and mechanical properties are of the essence.

## 6. Implementing Capacitors in Flexible Designs

Recently, flexible capacitors that translate a change in strain into a change in capacitance have emerged [172,173]. These can be a useful alternative to lumped elements, given their robust mechanical integration. However, their fabrication and incorporation into the sensor can be complex.

### 6.1. Small Form Integrated Capacitors

Embedded passive technology (such as capacitors and inductors) in substrates has been crucial to improve mechanical stretchability while maintaining RF performance. First and foremost in this regard is the importance of constructing flexible small-form capacitors with adequate electrical performance, which are able to withstand extensive deformation.

**Figure 6:**
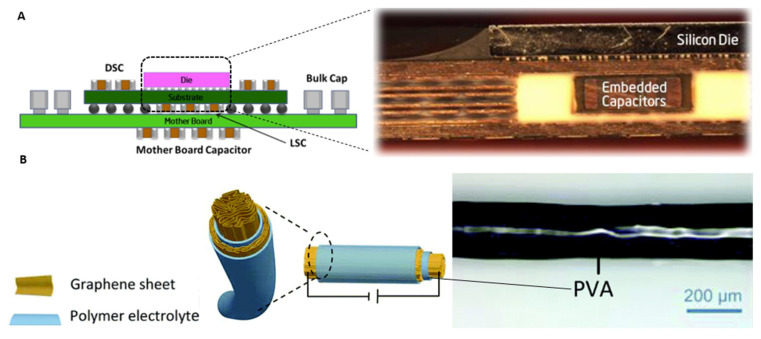
(**A**) Schematic cross-sectional view of embedded capacitor components on a motherboard [175]. (**B**) Illustration of polyvinyl-alcohol gel-coated axial graphene fiber supercapacitor [177].

The main function of a capacitor is to absorb energy in the form of an electric charge. The available size of this capacitive charge depends on the material composition and geometric characteristics [174]. Therefore, substrate thickness plays a role in the capacitances available to a sensor design. One approach toward the miniaturization of capacitor designs has been to reduce the thickness of the dielectric layer and thus the dimensions of the capacitor (see (Figure 6A)) [175]. In this realm, capacitors have been embedded in ceramic substrates among other things [176].

### 6.2. Integration of Capacitors in Fabric-Based Substrates

A second requirement is the integration of these capacitors in the substrate, often achieved via electromechanical designs. Some of these methods have proven challenging, especially in the case of textile substrates. For instance, graphene- and carbon-based fibers (Figure 6B) have led to porous and limited mechanical stability of textile-woven capacitors [177,178]. Other energy storage designs, such as harvester systems, have been shown to lack sufficient power density [179,180]. However, Meng et al. developed sheath-core graphene-based fiber supercapacitors that are highly compressible, stretchable, and weavable into a textile [181]. Nevertheless, there is a strong need for the development of new high-performance electrochemical energy storage modalities that can be easily integrated with wearable electronics.

Table 4 presents the electromechanical properties of the flexible capacitors described in the text. The various capacitor designs exhibit different electromechanical parameters, mainly due to inherent differences in the geometric microstructure of their materials, such as textiles, gels, thermoplastics, and ceramics. In general, a higher tensile strength is desired, but it can come at the cost of available energy density. Another factor to be considered when choosing an appropriate capacitor design is the available power density. 

## 7. Fabrication Methods

**Figure 7:**
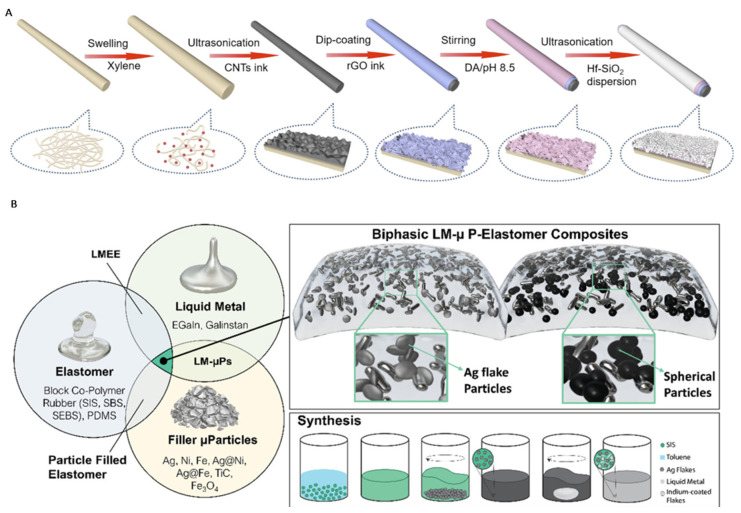
(**A**) Schematic of a complex fabrication process for conductive fibers [182]; (**B**) illustration on the assembly of liquid metal binary and ternary composites [183].

Fabrication methods for the integration of substrates with conductive inks range from screen printing, embroidering, and wet spinning to ultrasonication. An illustration of various multi-step fabrication processes can be seen in Figure 7A [126]. Many smart materials and textiles have been adapted with built-in electrodes to provide functional “smart” clothing. Despite the impressive progress in these fabrication methods, there is still a need for mass-producible streamlined fabrication to integrate electronics with clothing material [69].

### 7.1. Maintaining Mechanical and Electrical Performance of Different Fabrication Techniques

First, the effect of different fabrication methods on electrical properties has to be considered. Folded or twisted liquid metal fiber tubing systems have shown some variation in electrical resistance [184,185] when prepared via a traditional spinning method, thereby affecting conductivity by disrupting the electrical connection within the fiber.

Second, the mechanical properties of the sensor need to be maintained throughout fabrication. Many of the textile sensors, while impressive and innovative, suffer from adhesion issues, requiring heat seal techniques or other complex methods to attach TPU or other materials onto the fabric [186,187]. Exposure to these high temperatures tends to deteriorate the textile fabric.

Ultimately, only a limited number of studies have focused on fabrication methods that maintain the critical tradeoff between stretchability and electrical performance [188].

### 7.2. Screen Printing

Thus far, screen printing has been the most common method used to implement electrodes in substrates as it is affordable, easy to handle, and repeatable [189]. A few parameters must be considered with this technique, such as the need to print directionally (0°, 45°, 90° degrees) and the need to consider the pattern structure of the fabric when using certain electrode geometries. These design constraints ultimately determine the performance of the electrode fillers. The orientation of fabric threads should coincide with the printing direction. Annular geometric electrode structures result in nonuniform electrode patterning [190] because they do not follow fabric directionality. The importance of the microstructure inherent to textiles is highlighted in [144]—conductive ink and printing features are crucial factors that impact sensor assemblage. Not all fabrics can be screen-printed due to their absorption capabilities, thread diameter, and textile voids [144].

### 7.3. Three-Dimensional Printing

Recently, 3D printing has emerged as the most reliable and suitable fabrication technique due to its affordability, reproducibility, and repeatability. Both the substrate materials and the conductive inks can lend themselves to 3D printing techniques. In particular, printing ink, usually conductive, in this manner has found popularity in a method called direct ink writing (DIW).

A few groups have begun to explore biphasic composites to improve 3D printing performance. The styrenic block copolymer-based resins mentioned earlier for their adhesive qualities, electrical conductivity, and favorable electrode patterning [145] also possess formidable compounding and viscoelastic characteristics necessary for successful 3D printing. Other groups, such as Wang et al., reported to have 3D-printed a sheath-core fiber consisting of a styrene–ethylene–butylene–styrene (SEBS) shell with a GaInSn alloy core. The conductive fiber was successfully printed directly onto the fabric [191]. The fiber achieved stretchability of up to ~600% and comparable conductivity to conventional copper wire. However, it is important to note that such styrene composites have been shown to lack repeatability under mechanical deformation [142,143]. Further, SEBS conductive inks have been shown to be incompatible with traditional 3D printers. Structural destabilization of styrene-based ink is common, and print defects are present, as SEBS must be heated to high temperatures < 180 °C (above the melting point of the ink) to be extruded. Manufacturing methods, therefore, need to be revised continuously before implementing block copolymer composites in existing conductive inks. Another recurring issue is temperature annealing, which can pose a degradation issue as high temperatures can directly degrade textile fabrics. Hence, parameters such as printing speed, nozzle diameter, and printing temperature must be evaluated simultaneously to ensure smooth trace patterning and ink deposition. The need for a conductive ink that can be 3D printed with a continuous trace pattern on a substrate is pivotal to achieving a highly reliable wearable sensor.

Recently, a sinter-free liquid metal-Ag-SIS composite was fabricated using DIW [183]. The ternary conductive composite ink incorporated additive materials, such as nickel, iron, ferrite, and titanium carbide. An illustration of the assembly of ternary composites was reported (Figure 7B). The study investigated the role of additive filler material and reported that the choice of materials ultimately influences the rheological properties of liquid metal biphasic conductive inks. A comprehensive review of recent advancements in biphasic composite inks using liquid metal is provided in [135]. Ultimately, liquid metal-based composites have yet to be incorporated into clothing to form an autonomous sensor [69]. Many conductive inks still require the incorporation of additive materials. Researchers in [141] reported having successfully 3D-printed on non-woven textiles using a sodium alginate natural resin biphasic liquid metal composite. As shown throughout the study, liquid metal–solid biphasic composites offer formidable electrical performance for many sensor applications compared to simple liquid metal.

Ultimately, an in-depth consideration of filler material composites and reaction kinetics is required when choosing the most suitable material for a sensor device.

### 7.4. Fabrication Methods for Capacitor Integration

Textile and polymers offer many benefits when used as dielectric substrates. Among these is an inherent frequency stability, leading to a stable capacitance. A few substrate-inherent limitations, such as its thickness and dielectric permittivity, have to be taken into account when implementing integrated capacitors [174]. As a result, it is important to identify fabrication methods that can provide tailored substrate thickness.

#### 7.4.1. Methods for Controlling Substrate Thickness to Accommodate Capacitors

A limited number of studies have examined the integration of polymeric components in fabric-based substrates [192] with a specific focus on better control of device thickness. Repeatability in sensor thickness has posed a challenge when using textiles as substrates. The printing of conductive inks (e.g., graphene, carbon nanotubes, Ag, and nickel) on non-woven fabrics requires special surface deposition such as doping or coating to protect the electrode from external excessive friction, tearing, and degradation from wash cycles [193,194].

Both screen printing (yielding substrate heights of up to 100 μm) and inkjet printing (heights of up to 0.6 μm) provide tailored substrate thickness. However, both methods are prone to the smudging of ink, as illustrated in Table 5. Aerosol jet printing provides small-size droplets for good printing resolution, but overspraying defects are present [195]. DIW is prevailing as an ideal printing approach to polyester textiles as it provides the highest DIW precision, reasonable resolution, as well as affordability, and avoids the need for additional high-temperature sintering [196]. State-of-the-art printing technology specifications are summarized in Table 5.

#### 7.4.2. Fabrication Techniques Suited to Capacitors Integrated in Fabrics

Although the field has made significant progress, many of the substrates, such as textiles, are still difficult to be printed on. Thus far, the fabrication methods for capacitor integration in textile sensors consist of sputtering (i.e., vapor deposition) [199], gluing [200], sewing [201], embroidery [202], stitching [201], doping/coating, knitting, and 3D printing [156].

## 8. Future Improvements Specific to RF Coils Based on Flexible Sensor Technology

To recall, the main challenge inherent to MRI is the limited SNR of rigid commercial coils designed to accommodate larger anatomies. The resulting increased distance between the coil and tissue significantly impacts signal performance, leading to suboptimal SNR. Flexible and stretchable coils are capable of providing a conformal fit, and bendability is essential for achieving optimal SNR.

Customizing sensor materials and manufacturing principles allows for innovative stretchable coil designs. The continuation of combining existing material composites with solid-phase materials could improve imaging performance and aid diagnostics. However, a few considerations specific to the constraints encountered in MRI are needed. Current MRI-specific areas of investigation thus include frequency detuning, signal loss from metal artifacts and lossy materials, as well as array-specific fabrication.

Knowing and understanding the electromagnetic material characteristics at the Larmor frequency, not always in the vicinity of other sensor frequency bands, is instrumental in the adequate pairing of materials. Further knowledge of the mechanical characteristics can provide different options for form-fitting designs capable of conforming to anatomical curvature and variations in patient size. It is also crucial to optimize fabrication techniques. Further exploration of biphasic material additives can mitigate frequency detuning from mechanical hysteresis.

In the specific case of textile RF coils, the rapid disillusionment of these materials must be examined because frequency detuning and lack of long-term functionality can ensue. Therefore, understanding the microstructure of textile materials and the interfacial behavior of the woven fiber fabric is paramount, as is their compatibility with conductive inks.

**Figure 8:**
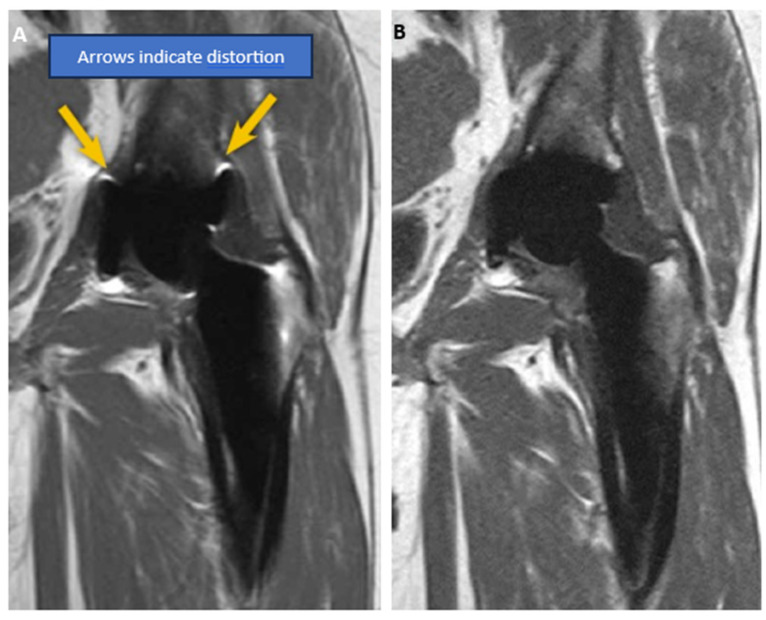
Same patient’s MR images of conventional hip metal implant: (**A**) metal-induced artifact present in MR image; (**B**) no distortion present in MR image [204].

Diminishing signal strength and, in turn, resolution are incurred when using hard and soft material composites [203]. The presence of certain material composites in MRI such as magnetic metals (e.g., iron and cobalt) and long wires (e.g., copper) have been shown to cause image degradation, as seen in Figure 8A,B. Another crucial aspect lies within the manufacturing of RF coil arrays, where several layers, each containing a single coil element, must be overlapped in a predefined geometric way to form the array.

Focusing on areas of research centered around material development can guide future advances in sensor science. For instance, optimizing material combinations will enable better mechanical properties and electrical performance. The tradeoffs that need to be found when using such materials in wearables usually contrast mechanical strength/flexibility and electrical performance and include substrate thickness, weak conductor adhesion, geometric confinement, impurities in material microstructure, and external electromagnetic configurations.

The development of appropriate capacitor implementation will also aid electrical performance. Here, small-form lumped components with highly specific dielectric permittivities are needed, which stands in contrast to the less stringent geometric and dielectric requirements of substrates per se and their more urgent mechanical deformability.

Improvement of fabrication methods can enable large-scale production of sensors in RF coil systems. Often, a lower cost is desired in fabrication methods, which can come with some of the undesired effects observed in techniques such as screen printing, requiring highly viscous inks, inadequate substrate thickness controllability, and forming high-resolution uniform patterns.

Providing stretchable coils for MR imaging delivers a highly useful diagnostic benefit; however, sufficient imaging performance and adequate biocompatibility as well as sturdiness of the end product can be of much higher importance in clinical practice.

Ultimately, combining knowledge from several research arenas such as material science, electromagnetics, radiology, and fabrication technologies can provide an in-depth understanding of sensor and diagnostic mechanisms. A careful weighing of different advantages and drawbacks can result in a product that can significantly contribute to advancing the field of medical imaging and radiology hardware.

Our group has overcome some of these existing technical challenges in flexible electronics specific to MRI coil design. For instance, we have found that we can meet the bendability and form-fitting stretchability requirements of complex anatomies when combining elastomer and liquid metal technology [8,13]. Further, our group showed that we can integrate a smart interdigital capacitor that changes its capacitance in response to the inductance change from stretching, which provides a coil with a stable resonance frequency and thus stable SNR [8]. We have also shown 2D stretching without frequency shift [21]. Some of the silicone-based elastomer materials were visible in the MR images, and we have been able to dope the material with Magnevist to reduce its MR signal [20]. Further, we have provided direct ink writing fabrication of these coils because it becomes difficult to form and, specifically, to accurately and geometrically de-couple multi-element arrays from handmade single-element coils [13]. Each of these prototypes has provided adequate MR signal and imaging performance, and, in particular, improved SNR over rigid commercial coils that sit far away from the anatomy.

The key takeaway from this comprehensive exploration of existing technology is that while significant progress has been made, further investigation into materials and manufacturing may be useful to maximize the potential of flexible sensor technology. Potential improvements in sensor substrates and conductors include the identification of material combinations that mitigate extensive load deformation, lack of adhesion, deterioration in electrical performance, durability, sustainability, biocompatibility, cost-effectiveness, fabrication scalability, and reliability.

## 9. Conclusions

This review begins by summarizing the existing body of research in stretchable MRI RF coil designs. It then discusses existing materials used in flexible sensor designs, categorized by substrates, conductors, and suitable combinations of the two. We also examined the implementation of lumped elements such as capacitors. We also discuss the MRI-specific requirements that distinguish stretchable coil designs from more conventional flexible sensors.

The development of wearable sensors and stretchable MRI coils is at the forefront of the demands in medicine and beyond. The continuation of this research, especially in the form of multidisciplinary exploration, is expected to deliver future breakthroughs. Ultimately, this promises improved diagnostics in MRI and optimized sensing performance elsewhere.

## Figures and Tables

**Table 1:** Comparative analysis of conventional fabric dielectric textile substrate materials.

Fabric Material	$\epsilon_{r}$	L*W	*δ*	Feed Point (mm)	*S*_11_	Gain (dBi)	*η*	BW (Return Loss)	*Z*	Ref.
Cotton	1.6	46*53	0.0400	13.5	−32	3.8	35%	0.097	48 − j0.789	[86]
Polyester	1.9	43*50	0.0045	8.0	−35	6.8	76%	0.040	48 − j1.849	[86]
Cordura	1.9	43*50	0.0098	9.5	−29	5.9	64%	0.050	50 − j2.450	[86]
Lycra	1.5	48*54	0.0093	9.0	−31	6.8	67%	0.048	47 − j0.740	[86]
Wool	-	-	0.0460	-	-	-	-	-	-	[87]

$\epsilon_{r}$ = relative permitivity; *δ* = Loss Tang; *S*_11 =_ reflection coefficient; *η* = efficiency; *Z* = impedance.

**Table 2:** Comparison of conventional and flexible conductor materials [94,95].

Conductive Materials	Conductivity (σ: S/m)	Ref.
Silver (Ag)	6.3 × 10^7^	[96]
Copper	5.98 × 10^7^	[96]
eGaln Liquid	3.4 × 10^6^	[97]
Polyurethane Nanoparticle Composite	1.1 × 10^6^	[98]
Zoflex + Copper	1.9 × 10^5^	[99]
Ag Flakes Fluorine Rubber	8.5 × 10^4^	[100]
AgNW/PDMS	8.1 × 10^5^	[101]
PANI/CCo Composite	7.3 × 10^3^	[102]
Copper Coated Taffeta	3.4 × 10^6^	[103]
Graphene	33 × 10^3^	[94]
eGaIn + Ag nanoparticles	6.38 × 10^5^	[104]
Carbon nanotubes	10^6^–10^7^	[105]

**Table 4:** Material pairing and capacitor performance from integrated capacitors.

Type of Capacitor	Electrode Filler	Dielectric Substrate	Tensile Strength	Energy Density	Power Density	Ref.
e-textile	Ag-plated copper wires	Cotton textile	-	-	-	[174]
Muli-layer	Nickel paste	BaTiO_3_	-	-	-	[175]
Supercapacitor	Carbon fibers	Woven Fabric	1955 MPa	3.6 mWh cm^−3^	>100 W/kg	[178]
Energy harvester	Copper	FR-4	15–20 MPa	-	1.07 mW cm^−3^ g^2^	[179]
Supercapacitor	Graphene/H_2_SO_4_-PVA gel	Woven textile	180 MPa	0.4–1.7 × 10^−7^ Whcm^−2^	6–100 × 10^−6^ W/cm^2^	[181]

**Table 5:** Comparison of different printing methods.

Methods	Ink Viscosity (cp)	Nozzle Diameter (μm)	Size (μm)	Thickness (μm)	Features	Ref.
inkjet printing	1–30	20–60	20–100	0.6	low cost, multiple heads	[197]
aerosol jet printing	1–1000	150–300	10–200	0.1	high throughput, thin layers, good features	[197]
direct ink writing	1–1,000,000	0.1–1	1–1000	0.5	highest precision, best resolution	[197]
screen printing	4000–12,000	N/A	N/A	10–100	wide shape and size, fragile manufacturing, soft stencils	[198]

## Data Availability

The data presented in this study are available upon reasonable request from the corresponding author.
